# Formation of iron oxide-apatite deposits triggered by magmatic assimilation of evaporitic sulfate

**DOI:** 10.1038/s41467-026-75189-0

**Published:** 2026-07-07

**Authors:** Stefan T. M. Peters, Dingsu Feng, Valentin R. Troll, Andreas Pack, Fernando Tornos, Ulf B. Andersson, Bernd Lehmann, Tommaso Di Rocco

**Affiliations:** 1 Leibniz-Institute for the Analysis of Biodiversity Change (ZBM – Mineralogy), Museum der Natur Hamburg, Grindelallee 48, 21046 Hamburg, Germany; 2 Georg-August-Universität Göttingen, Department of Geochemistry and Isotope Geology, Goldschmidstraße 1, 37077 Göttingen, Germany; 3 University of Uppsala, Department of Earth Sciences, Section for Natural Resources & Sustainable Development, Villavägen 16, Uppsala, 75236 Sweden; 4 Instituto de Geociencias (IGEO, CSIC-UCM), Dr Severo Ochoa, 7, 28040 Madrid, Spain; 5 University of Uppsala, Department of Earth Sciences, Section for Mineralogy, Petrology and Tectonics, Villavägen 16, Uppsala, 75236 Sweden; 6 Technical University of Clausthal, Adolph-Roemer-Str. 2a, 38678 Clausthal-Zellerfeld, Germany

**Keywords:** Geochemistry, Petrology, Volcanology, Element cycles

## Abstract

The geological origins of iron oxide-apatite (IOA) rocks, important resources for iron and rare-earth elements, are intensely debated. Using triple oxygen isotope data, we here show that magnetite from IOA deposits near Kiruna, northern Sweden, and related igneous rocks contain high concentrations of oxygen derived from evaporitic sulfate. To explain these observations, we propose that the Kiruna IOA assemblage formed in response to massive assimilation of evaporites by silicate magmas. Sulfate from the evaporites would have oxidised ferrous iron in these magmas, facilitating the formation of immiscible ferric iron-rich melts and/or magnetite, which then separated from the magmas to form ore deposits. Ferric iron-bearing fluids with low Δ′^17^O values, exsolved from the silicate magmas or the ore-forming melts, would have crystallised additional magnetite. An inventory study reveals that Proterozoic and Cambrian IOA deposits have lower Δ′^17^O values than post-Cambrian IOA deposits. This shows that the Δ′^17^O values of global IOA deposits reflect the changing isotope composition of atmospheric O_2_ incorporated by evaporitic sulfate over time, and demonstrates that oxygen released from evaporitic sulfate is a common component in IOA deposits.

## Abstract (web summary)

This study uses triple oxygen isotope analyses to demonstrate that iron oxide-apatite (IOA) deposits, including those in the Kiruna district of Sweden, contain significant amounts of oxygen derived from evaporitic sulfate, indicating direct involvement of assimilated evaporites in their formation. The findings suggest that magmatic assimilation of evaporitic sulfate triggered oxidation processes that facilitated IOA mineralisation, and that the oxygen isotope signatures in IOA deposits globally reflect changes in atmospheric oxygen over geological time.

## Introduction

Iron oxide-apatite (IOA) deposits, also known as magnetite-apatite or Kiruna-type deposits, are phosphorus-fluorine-rich, magnetite-dominated rock assemblages frequently mined for iron. Despite over a century of research^1^, the geological processes leading to the formation of IOA deposits remain poorly understood. Most proposed formation models involve either igneous or hydrothermal processes, or a combination of both^2^. Suggested igneous formation processes include the formation of magnetite and apatite from iron oxide melts that exsolved from iron-rich silicate magmas^3–9^ and/or from iron-rich sulfate-carbonate melts^10–13^. In contrast, proposed hydrothermal formation processes involve magmatic-hydrothermal fluids or the dissolution, transportation, and reprecipitation of iron by aqueous fluids derived from magmatic and/or meteoric sources^14–16^.

Many IOA deposits formed in association with magmatic intrusions and/or fluid circulation in evaporite-rich sediments^10–13,17–23^. However, the potential significance of evaporites in the formation of IOA deposits has not been thoroughly investigated. To test whether evaporites were not only associated with but also involved in the formation of IOA deposits, we examined whether evaporite-derived oxygen can be traced in magnetite of IOA deposits by studying their triple oxygen isotope ratios. A useful and descriptive parameter for studying triple oxygen isotope ratios is Δ′^17^O, which we define here as:

$$\Delta^{\prime{17}} {{\rm{O}}}/1000 = 1000 \, {{\rm{ln}}} \, ({\updelta}^{17} O/1000 + 1) - 0.528 \times 1000 \, {{\rm{ln}}} \, ({\updelta}^{18} {{\rm{O}}}/1000 + 1)$$

The parameter Δ′^17^O is a potential tracer for evaporite-derived oxygen in rocks, because evaporitic sulfate can carry a component of oxygen that was derived from atmospheric O_2_^24,25^. Atmospheric O_2_ has a significantly lower Δ′^17^O than most rocks and fluids, owing to a mass-independent isotope effect that is associated with the formation of stratospheric ozone, and to kinetic isotope effects that are associated with the Dole effect^26,27^. The parameters that govern the Δ′^17^O of atmospheric O_2_, e.g., atmospheric *p*CO_2_ levels and gross primary productivity of the biosphere, have varied throughout Earth’s history^27,28^. As a result, evaporites of Proterozoic and Cambrian ages can have Δ′^17^O values down to more than minus a thousand ppm, whereas more moderate Δ′^17^O values are recorded by post-Cambrian evaporites^25^. The sensitivity of the parameter Δ^′17^O as a possible tracer for evaporite-derived oxygen in rocks, therefore, varies with the age of the rocks studied.

Much different than atmospheric O_2_ and evaporitic sulfate, most crustal rocks, minerals, and fluids plot along an array in Δ′¹⁷O–δ¹⁸O space (a terrestrial fractionation array or TFA)^29,30^. When interpreting triple oxygen isotope data of rock samples, this TFA provides a baseline for assessing whether rocks contain evaporite-derived oxygen or not. The δ¹⁸O values of most magnetite samples from IOA deposits plot in an interval of the TFA with δ¹⁸O values of ca. –1 to 5‰^3,14,17,31–33^, for which the corresponding Δ′¹⁷O range is poorly constrained. When comparing the triple oxygen isotope compositions of rock samples with the TFA, we therefore rely on the Δ′¹⁷O values of rocks and minerals with slightly higher δ¹⁸O values, i.e., between 5 and 10‰. These rocks typically have Δ′¹⁷O values between –75 and –45 ppm^29,30,34^. Fluids with lower δ¹⁸O values than the samples studied here, in contrast, have higher Δ′¹⁷O values than –45 ppm^29^. Thus, the IOA rocks studied are considered anomalously low relative to the TFA if they have Δ′¹⁷O values below –75 ppm.

First, we conducted a case study of the Paleoproterozoic type locality of IOA deposits: the 1.9 Ga-old rocks from the Kiruna mining district in northern Sweden^35^. We determined the triple oxygen isotope compositions of 22 magnetite samples and apatite samples from five different mines in the Kiruna region (Kiirunavaara, Rektorsgruvan, Mertainen, Nukutusvaara, Luossavaara; Supplementary Data 1). In order to investigate the relation between the IOA deposits and their associated igneous porphyry rocks, we also determined the triple oxygen isotope compositions of magnetic and non-magnetic mineral fractions separated from nodular porphyry rocks from Mertainen, Luossavaara, and Kiirunavaara, and from magnetite-syenite porphyry rocks from Luossavaara, and Kiirunavaara (Supplementary Data 2). The magnetic fractions from these samples consisted solely of magnetite, while the non-magnetic fractions contained various silicate phases and minor titanite. We then performed an inventory study of magnetite samples from other Proterozoic IOA deposits, including those of the Paleoproterozoic Malmberget and Grängesberg mining districts (1.9 Ga) in northern and central Sweden, respectively, the Paleoproterozoic Vergenoeg fluorite-magnetite deposit in the Bushveld Complex of South Africa (2.0 Ga), the Mesoproterozoic iron ore province of southeast Missouri (Pea Ridge, Iron Mountain, Pilot Knob; 1.4 Ga), and the Mesoproterozoic iron ore deposits from the Adirondacks mountains (High Ledge mine, Skiff mine; 1.0 Ga). In order to investigate whether evaporite-derived oxygen can also be traced in post-Cambrian IOA deposits, we additionally studied samples from, respectively, the deposits of the Chilean Iron Belt (150–130 Ma) that are predominantly of Jurassic-Cretaceous ages, the Paleogene Cerro de Mercado deposits in Mexico (31 Ma), and the Quaternary deposits of El Laco, Chile (2 Ma). For comparison, we also determined the triple oxygen isotope compositions of magnetite from the Grasberg porphyry copper-gold deposit in Indonesia (3 Ma), a Cretaceous strata-bound ore body from the Chilean Iron Belt (Bandurrias), two Neoarchean iron-oxide-copper-gold deposits (Salobo, Sossego; Carajás mineral province, Brazil), and one Mesoproterozoic nelsonite sample from Port Leyden, Adirondacks Mountains (USA).

## Results

**Fig. 1:**
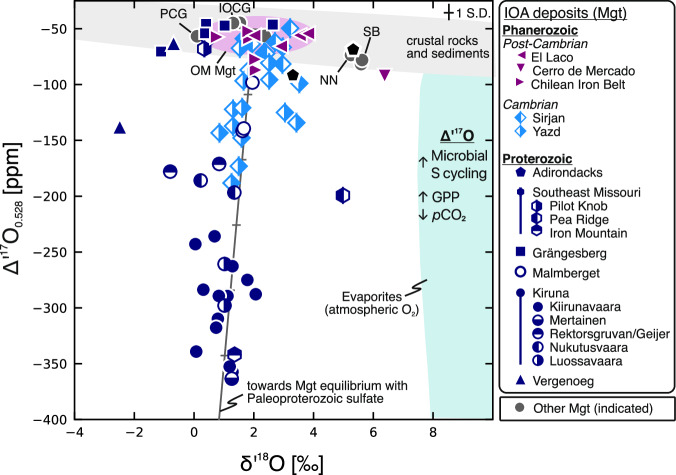
Δ′^17^O versus δ′^18^O in magnetite from Kiruna and other IOA deposits. Listed and colour-coded by age, and compared to the compositions of common crustal rocks and sediments^29,30,34,37^ and ancient evaporites^25^. Also shown are data for magnetite from iron oxide copper gold (IOCG) deposits, a porphyry copper gold deposit (PCG), a strata-bound orebody (SB), a nelsonite sample (NN), and the suggested composition of (ortho)magmatic magnetite (OM Mgt)^17^. Magnetite compositions that plot below the range of crustal rocks and sediments are interpreted to contain oxygen from ancient evaporitic sulfate in addition to oxygen from a magmatic source. For illustration, mixing is shown between a typical composition of magmatic magnetite and a hypothetical magnetite end member composition that is in equilibrium with evaporites that formed between 2000 Ma – 1100 Ma (“Mid-Proterozoic” era as defined by Crockford et al. (2019)^25^; δ^18^O = 8.49 ‰; Δ′^17^O = -635 ppm). Arrows in the field for “evaporites” indicate the directions in which sulfate Δ′^17^O values have shifted throughout Earth’s history with varying atmospheric *p*CO_2_ levels and gross primary productivity (GPP) of the biosphere; as well as with sulfur cycling processes^25,27,28^.

The magnetite samples from the IOA assemblage at Kiruna have Δ′^17^O values that range from −171 ppm down to −363 ppm (Supplementary Data 1), and therefore have among the lowest Δ′^17^O values that were detected in any igneous and hydrothermal minerals so far^29,30,34,36,37^ (Fig. 1). The coexisting apatite from the IOA assemblage is fluorapatite and has anomalously low Δ′^17^O values as well (ranging from −149 ppm down to −285 ppm, Supplementary Data 1, 3). Four out of six of the magnetite fractions of the porphyry rocks from Kiruna have anomalously low Δ′^17^O values as well, ranging down to −341 ppm for a syenite porphyry from Kiirunavaara (Supplementary Data 2). The non-magnetic mineral fractions tend to have more moderate Δ′^17^O values than the coexisting magnetite fractions, but with three to four of the non-magnetic fractions having lower Δ′^17^O values than typical igneous rocks.

Similar to Kiruna, magnetite samples from Malmberget, Vergenoeg and from two deposits in southeast Missouri (Iron Mountain and Pea Ridge) show anomalously low Δ′^17^O values, whereas magnetite from a third deposit in southeast Missouri (Pilot Knob) has a similar Δ′^17^O as most crustal rocks. For the Adirondacks Mountains, a sample from Skiff Mine has a low Δ′^17^O value, whereas a sample from High Ledge Mine has a Δ′^17^O similar to most crustal rocks. Five out of six magnetite samples from the Grängesberg mining district have Δ′^17^O values similar to crustal rocks, and the sixth sample has a low Δ′^17^O value. Magnetite samples from the Chilean Iron Belt show a tendency towards lower Δ′^17^O values, but Δ′^17^O values from only one of the deposits (Carmen) can be resolved from typical crustal rocks. Magnetite from Cerro de Mercado has a low Δ′^17^O as well. Our magnetite and apatite samples from El Laco have Δ′^17^O values similar to crustal rocks, but magnetite samples from El Laco with lower Δ′^17^O values around ca. −100 ppm have been reported^31^. Samples from other types of iron deposits that we investigated have Δ′^17^O values overlapping with the compositions of typical crustal rocks and sediments, at the given δ^18^O values of the samples.

## Discussion

The exceptionally low Δ′^17^O values in the magnetite and apatite samples from Kiruna cannot be explained by mass-dependent oxygen isotope fractionation processes^37,38^. Instead, a possible explanation could be if a component of ancient atmospheric O_2_ with mass-independently fractionated oxygen is present in the samples (Fig. 1)^17^. The most plausible source for atmospheric oxygen in the samples is evaporitic sulfate – the only known type of geological material that has captured large quantities of oxygen with anomalously low Δ′^17^O from the atmosphere^24,39^. The regional occurrence of scapolite in the area of the IOA deposits in the Kiruna district^40,41^ is in good agreement with the suggestion that evaporites were possibly associated with the deposits, and so are the suggestions that the host rocks of the deposits formed in a continental back-arc extensional environment^42,43^ and/or as caldera fillings^44^.

**Fig. 2:**
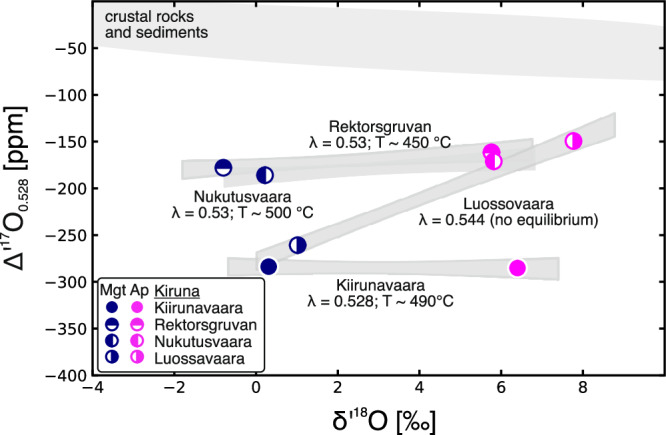
Triple oxygen isotope compositions of coexisting magnetite and apatite in samples from Kiruna mines. Three of the samples plot on apparent fractionation slopes with λ-values that are consistent with mass-dependent isotope fractionation^54^. Apparent equilibrium temperatures calculated from ^18^O/^16^O fractionation between magnetite and apatite are indicated^55,56^, and are interpreted to reflect isotopic re-equilibration between magnetite and apatite with fluids in a rock‑buffered system or, alternatively, by solid‑state diffusion, during upper greenschist facies metamorphism. Such conditions for isotopic exchange would not have significantly modified the Δ′^17^O values of the samples.

It is important to determine whether the low-Δ′^17^O component in our magnetite samples reflects primary mineralisation or a post‑mineralisation metamorphic overprint. Magnetite-apatite pairs in three samples from the Kiruna IOA assemblage record oxygen isotope equilibrium temperatures of 450 – 500 °C (Fig. 2). For Kiruna, this temperature range is similar to previously reported greenschist facies conditions (T = 300 °C – 450 °C) to which the IOA assemblage was exposed several millions of years after ore formation^45^. Moreover, several much younger episodes of hydrothermal overprints have been documented in the area^46,47^. The δ^18^O values of magnetite and apatite in these particular samples were therefore possibly modified by isotopic exchange between magnetite and apatite during metamorphism or later hydrothermal events unrelated to the mineralisation process. Importantly, such exchange could have involved external fluids^45^, raising the possibility that the anomalously low Δ′^17^O values of our samples reflect equilibration with an evaporite-derived metamorphic fluid at high fluid/rock ratios, rather than showing a primary signature of mineralisation. We consider this scenario unlikely, because magnetite-apatite pairs from three different Kiruna mines yield indistinguishable equilibrium temperatures within <50 °C. Fluid‑rich metamorphism, in contrast, would be expected to produce spatially heterogeneous temperatures reflecting variable fluid access and flow paths. If our magnetite-apatite pairs would indeed record equilibrium temperatures of metamorphic oxygen isotopic exchange, they would therefore need to have either exchanged with fluids in a rock‑buffered system or, alternatively, by solid‑state diffusion only. Neither scenario would have significantly altered the Δ′¹⁷O values of magnetite and apatite by more than ~20ppm from their pristine compositions. We therefore conclude that the low Δ′¹⁷O values in our samples must represent a primary feature of the Kiruna IOA mineralisation process, rather than a metamorphic or hydrothermal overprint. This interpretation is further supported by similarly low Δ′¹⁷O values in IOA samples from other localities that will be discussed below.

In order to explain the lowermost magnetite Δ′^17^O values from Kiruna (Δ′^17^O = −363 ppm), a substantial portion of oxygen of up to several tens of oxygen atom per cent in the magnetite samples must have been derived from evaporitic sulfate, given that typical Δ′^17^O values of Mid-Proterozoic evaporitic sulfate are ~ −635 ppm (i.e., the median Δ′^17^O for evaporitic sulfate that was deposited between 2.0–1.1 Ga in the compilation by Crockford et al. (2019)^25^, recalculated relative to a λ-value of 0.528). Therefore, we argue that evaporitic sulfate was not only associated with, but also directly involved in the magnetite mineralisation processes at Kiruna.

**Fig. 3:**
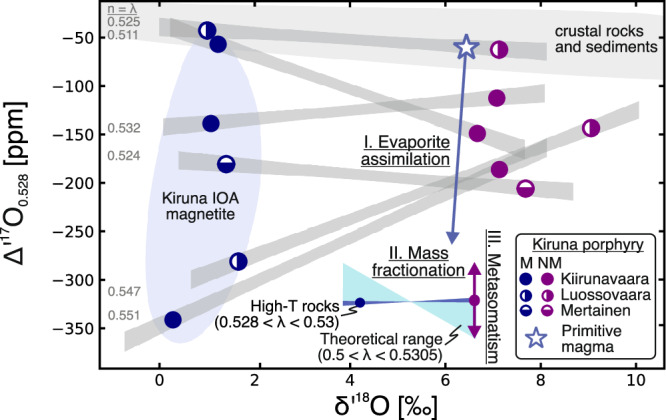
Δ′^17^O versus δ′^18^O in igneous porphyry rock samples from Kiruna. Samples from indicated mines were each processed into a magnetic (magnetite; M) and a non-magnetic fraction (various silicate minerals; NM). Shaded areas correspond to indicated λ-values of the samples; λ-values corresponding to mass-dependent oxygen isotope fractionation are shown for comparison in the inset. A simplified three-stage formation model of the porphyry rocks is illustrated: I. Mantle-derived magmas (star) assimilated evaporitic sulfate carrying the Δ′^17^O anomaly of Proterozoic air O_2_, resulting in parental magmas of the porphyry rocks with anomalously low Δ′^17^O values. II. Mass-dependent oxygen isotope fractionation during magma crystallisation led to the formation of magnetite with ~7 ‰ lower δ^18^O and similar Δ′^17^O values compared to coexisting silicates in the porphyry rocks. III. Metasomatic reactions, involving fluids with different Δ′^17^O values than the pristine porphyry rocks, led to the formation of secondary silicates, which resulted in the observed λ-values of the samples outside the range for mass-dependent oxygen isotope fractionation.

An important constraint on how evaporites could have been involved in the mineralisation process comes from the anomalously low Δ′^17^O values of the porphyry rocks from Kiruna, indicating that evaporitic sulfate-derived oxygen was also incorporated into igneous silicate rocks closely associated with the IOA assemblage (Fig. 3). We propose that the anomalously low Δ′^17^O values in the porphyry rocks are best explained if they formed from silicate parental melts that assimilated considerable amounts of evaporitic sulfate. To account for the lowest magnetite Δ′^17^O value in our sample suite through the magmatic assimilation of evaporites, up to ~50% of the magnetite oxygen atoms in the porphyry rocks would have originated from evaporitic sulfate, assuming that the sediments contributing to the magma had a Δ′^17^O typical for evaporites between 2000 – 1100 Ma (Δ′^17^O = − 635 ppm). We also explored whether, alternatively, the low Δ′^17^O values of the porphyry rocks could reflect metasomatic alteration of isotopically “normal” igneous rocks that reacted with anomalously low Δ′^17^O aqueous fluids exsolved during crystallisation of an iron-rich melt. In this scenario, however, metasomatic reactions at very high fluid/rock ratios of at least >5 are required to achieve the lowest Δ′^17^O values found in the porphyry rocks. At such high fluid/rock ratios, magnetite and silicate minerals would exchange oxygen isotopes with metasomatic fluids at near equilibrium conditions; but the magnetic and non-magnetic fractions of the porphyry rock samples, in contrast, are not in oxygen isotope equilibrium (Fig. 3). Thus, even though the oxygen isotope compositions of the porphyry rocks were metasomatically altered by fluids, metasomatic fluid/rock ratios were much more moderate than those required to explain the observed magnetite compositions. Given the presence of metasomatic minerals in the porphyry rocks such as albite, the oxygen isotope compositions of the non-magnetic fractions were likely the ones most affected by metasomatic alteration. We conclude that magnetite and, to lesser extent, silicate minerals in the porphyry rocks acquired their low Δ′^17^O values from their parental magmas having assimilated evaporitic sulfate.

The assimilation of evaporitic sulfate by silicate magmas could efficiently oxidise them. Evaporitic sulfate assimilated by silicate magmas would react with ferrous iron in association with, or possibly driven by, the degassing of SO_2_ (Reaction 1)^48^:

1 $${{{{{\rm{SO}}}}_{4}}^{2-}}_{({{\rm{evaporite}}})}+{3{{\rm{Fe}}}}^{2+}{{{\rm{O}}}}_{({{\rm{melt}}})}={{{\rm{SO}}}}_{2({{\rm{gas}}})}+{{{\rm{Fe}}}^{3 +}}_{2}{{{\rm{O}}}}_{3}.{{{\rm{Fe}}}}^{2+}{{{\rm{O}}}}_{({{\rm{melt}}})}+{{{{\rm{O}}}}^{2-}}_{({{\rm{melt}}})}$$

Given the large component of evaporite-derived oxygen in the IOA and porphyry rocks of Kiruna, we propose that assimilation of considerable volumes of evaporitic sulfate by iron-rich silicate magmas, and its associated oxidation of iron, could have promoted the formation of the IOA assemblage. The formation of SO_2_ gas by massive assimilation of evaporitic sulfate would additionally have facilitated the explosive degassing of the silicate magmas, similar to what is seen for El Laco^23,49^. Importantly, according to Reaction 1, magnetite would have crystallised with up to 4/7ths of the oxygen atoms derived from evaporitic sulfate. Our suggested formation reaction could therefore explain the lowermost Δ′^17^O values for magnetite from Kiruna, providing a plausible mechanism to quantitatively account for the evaporite-derived oxygen component in magnetite from the IOA assemblage.

**Fig. 4:**
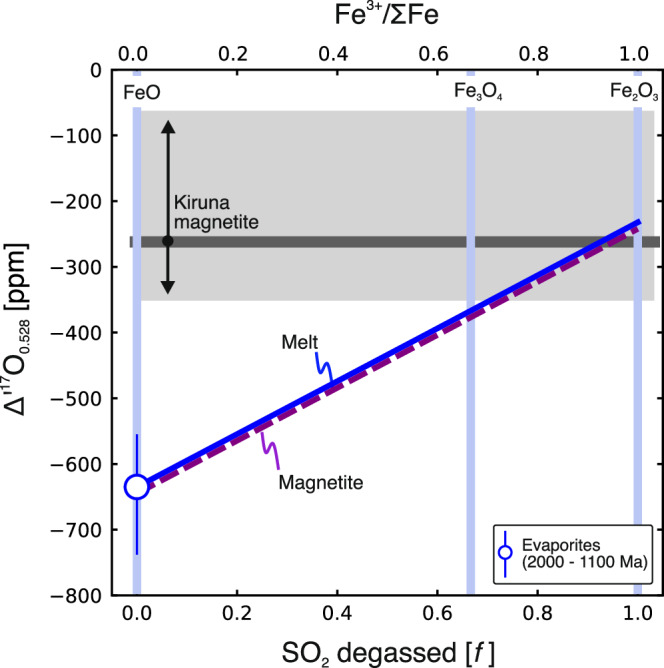
Modelled Δ′^17^O evolution of a hypothetical liquid derived from Proterozoic evaporites. The Δ′^17^O of a hypothetical liquid (solid line) that formed by melting of Proterozoic evaporites that were deposited between 2000–1100 Ma (open circle; error bars correspond to the P25 and P75 values^25^) increases as it scavenges FeO from magmas or fluids with a δ^18^O = 8 ‰ and a Δ′^17^O = −55 ppm. It is assumed in this particular model that the oxidation of FeO that was scavenged by the liquid drove the degassing of SO_2_ from the liquid (Reaction 1 in the main text), rather than vice versa. The Fe^3+^/ΣFe ratios are indicated for the entire system, i.e., for the sulfate liquid and FeO in silicate magmas, fluids, and/or rocks, collectively. Magnetite that would be in high-temperature equilibrium with the evolving hypothetical liquid is indicated with the dashed line. Magnetite Δ′^17^O values from Kiruna are shown for comparison (horizontal line = median value; field = all data), and serve to illustrate that the lowermost Δ′^17^O values in the sample suite could potentially be explained if the magnetite formed via the degassing of SO_2_ from the liquid.

The assimilation of evaporitic sulfate by silicate magmas could have triggered several end-member formation processes previously proposed to have led to IOA mineralisation. Primarily, reaction 1 may substantially have contributed to the formation of iron oxide-rich immiscible melts and thus the formation of IOA ore bodies^3–6^. This mechanism would be compatible with field observations from Kiruna suggesting that IOA rocks formed through crystallisation from (immiscible) fluid-rich melts^1,9,50–52^. Magnetite could additionally have crystallised directly from contaminated silicate magmas and then have concentrated to form ore bodies. Finally, the formation of anatectic sulfate-carbonate melts is consistent with our suggested geological formation setting as well, and could plausibly have produced magnetite with the Δ′^17^O range observed in the Kiruna samples (Fig. 4), aligning with evidence from fluid inclusions that such melts contributed to the formation of some IOA deposits^10–13^.

 For Kiruna, we propose that the assimilation of evaporitic sulfate by iron-rich silicate magmas formed the onset of high-temperature melt and fluid-driven mineralisation processes. The assimilation of evaporitic sulfate would have oxidised ferrous iron from the silicate magmas, enhancing the formation of magnetite and/or immiscible iron oxide melts that separated to form the ore bodies. Ferric iron-bearing fluids with low Δ′^17^O values, exsolved from these silicate magmas or the ore-forming melts, would have additionally reacted with the IOA assemblage and its associated magmatic rocks. These fluids then crystallised additional magnetite and apatite to form vein and disseminated ore types at lower temperatures.

**Fig. 5:**
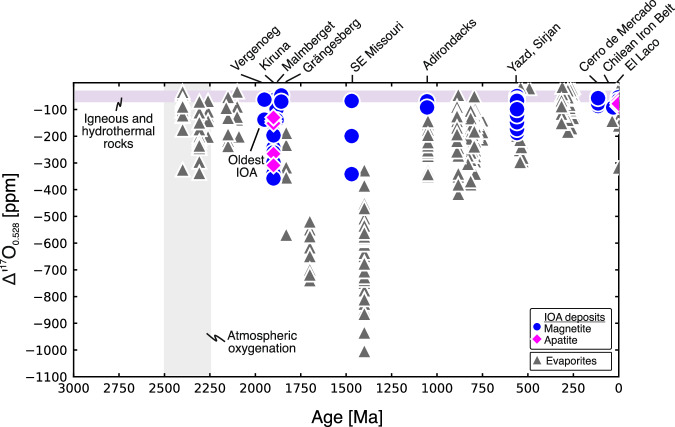
Δ'^17^O variations in IOA deposits through time. The Δ′^17^O values of magnetite (circles) and apatite (diamonds) from indicated IOA deposits are shown versus ages of the host rocks, and are compared to the compositions of evaporitic sulfate (triangles), and the age of atmospheric oxygenation^53^. Variations in the lowermost Δ′^17^O values of IOA deposits are interpreted to roughly reflect the varying Δ′^17^O of atmospheric O_2_ through time, i.e., similar to the trend that is seen in evaporitic sulfate. Magnetite data for Yazd and Sirjan are from Peters et al. (2020)^17^. Evaporite data are from the compilation by Crockford et al. (2019)^25^, and are recalculated relative to a λ-value of 0.528. The range for igneous and hydrothermal rocks comprises data from multiple sources^29,30,34,37^.

Magmatic assimilation of evaporites may be a common process that facilitated the formation of IOA deposits globally. Some of the magnetite samples from the Proterozoic IOA deposits of Malmberget, southeast Missouri, and Vergenoeg unambiguously contain significant amounts of evaporite-derived oxygen similar to Kiruna, and so do the Cambrian IOA deposits in Central Iran, as is demonstrated by their anomalously low Δ′^17^O values (Figs. 1, 5). The Proterozoic IOA deposits of the Grängesberg mining district do not show a resolvable evaporite-derived oxygen component, with the possible exception of one sample. In many evaporite deposits, however, the isotopically anomalous O_2_-derived oxygen component was erased by microbial sulfur cycling processes, without a significant effect in sulfate δ^18^O values^25^. Whereas the low Δ′^17^O values of magnetite from Kiruna, southeast Missouri, Vergenoeg, and possibly the Adirondacks, can trace evaporite-derived oxygen in these particular deposits, the “normal” Δ′^17^O values of the samples from the Grängesberg mining district, therefore, do not preclude an evaporite-derived oxygen component in those samples. Likewise, the magnetite samples from post-Cambrian IOA deposits show a more moderate range in Δ′^17^O values compared to Proterozoic and Cambrian IOA deposits, with indications for an isotopically anomalous oxygen component found only in some of these samples. This is an expected observation, however, if IOA deposits contain an evaporite-derived oxygen component, because post-Cambrian evaporites show more moderate Δ′^17^O values than Proterozoic and Cambrian evaporites^25^ (Fig. 5). In order to explain the collective dataset, we therefore propose that evaporite-derived oxygen could be a major component of many IOA deposits, with the lowermost magnetite Δ′^17^O values of the deposits reflecting the varying Δ′^17^O of atmospheric O_2_ through time (Fig. 5), in addition to sulfur cycling processes. Interestingly, the oldest known IOA deposits postdate the oxygenation of the atmosphere ~2.3 Ga ago^53^, a time prior to which sulfate-rich evaporites could not form. This observation strengthens our suggestion that IOA deposits in the rock record may be intimately linked to the occurrence of evaporite-rich sediments.

For other IOA deposits than those of the Kiruna district, additional sources of oxygen are possibly reflected in their δ^18^O values. Although the entire spread of magnetite δ^18^O values in the dataset could reflect the large range of δ^18^O values that is seen in evaporitic sulfate^25^, the elevated magnetite δ^18^O values of our samples from Pea Ridge (δ^18^O = 5.0 ‰), Cerro de Mercado (δ^18^O = 6.4 ‰), and some of the samples from Central Iran (uppermost δ^18^O = 6.8 ‰) compared to other IOA deposits may point towards a carbonate-derived oxygen component in these particular samples^17^. Extrapolating our formation model for Kiruna to these other IOA deposits would suggest that their formation was preceded by magmatic assimilation of both evaporites and carbonate rocks. The comparatively low δ^18^O value of one magnetite sample from Vergenoeg (δ^18^O = -2.5 ‰), in addition, could reflect the influence of meteoric fluids. Regardless of possible origins for the δ^18^O variations in our dataset, the recurrently low Δ′^17^O values in IOA deposits imply that evaporite-rich sediments likely played a pivotal role in the geological origins of IOA rocks.

## Methods

For triple oxygen isotope analysis of magnetite and the non-magnetic fractions of the porphyry rocks, similar protocols were followed as those in Peters et al. (2020)^17^. For the analysis of apatite, protocols identical to those in Feng et al. (2021)^36^ were used. Oxygen was extracted from magnetite, apatite, and silicate samples as O_2_ by means of laser fluorination, using BrF_5_ as the reagent. The sample gas was then purified from contaminant gases (e.g., BrF_x_ compounds, F_2_, NF_3_, N_2_) in an automated vacuum line at the University of Göttingen, using distillation techniques and a gas chromatograph^17,37^. Measured ^17^O/^16^O and ^18^O/^16^O ratios are reported in the conventional δ-values relative to VSMOW in per mill. All data are reported relative to San Carlos olivine with a Δ′^17^O value of -51.8 ppm (i.e., the average value from three independent studies^37^). In three samples, we also determined the major and trace element concentrations in apatite using a JEOL JXA-iHP200F electron probe microanalyser that is based at the Goettingen Laboratory for Correlative Light and Electron Microscopy (GoeLEM), University of Göttingen. Some of the magnetite and apatite samples from Kiruna, Grängesberg and El Laco were previously separated from bulk rock samples by Nyström et al. (2008)^3^ and Troll et al. (2019)^32^.

## Supplementary information

Description of Additional Supplementary Files

Supplementary Data 1

Supplementary Data 2

Supplementary Data 3

Transparent Peer Review file

## Data Availability

All triple oxygen isotope data for magnetite and apatite from IOA rocks, together with sample descriptions, are provided as Supplementary Data 1. Triple oxygen isotope data for the Kiruna porphyry rocks are listed separately as Supplementary Data 2. Apatite EMPA data are given as Supplementary Data 3.
