# Supplementary material for: Formation of iron oxide-apatite deposits triggered by magmatic assimilation of evaporitic sulfate
Source: Nat Commun. 2026 Jul 7;17:5930. doi: 10.1038/s41467-026-75189-0 (PMC13342607; doi:10.1038/s41467-026-75189-0)
Supplement: Supplementary file 1 — Description of Additional Supplementary Files [file 41467_2026_75189_MOESM1_ESM.pdf]

Description of Additional Supplementary Files

Supplementary Data 1|Triple oxygen isotope compositions of iron oxide apatite deposits and associated rocks

This table reports the $\delta^{17}O$ and $\delta^{18}O$ values of magnetite and apatite from the Kiruna district and other iron oxide–apatite (IOA) deposits, together with brief petrographic descriptions and sample locations. $\Delta'^{17}O$ values were calculated as $\Delta'^{17}O = 1000 [\ln(\delta^{17}O/1000 + 1) - 0.528 \times \ln(\delta^{18}O/1000 + 1)]$. All compositions are expressed relative to San Carlos olivine ($\Delta'^{17}O$ = −51.8 ppm; $\delta^{18}O$ = 5.33‰). For comparison, the dataset also includes magnetite from additional geological settings and ore deposits. All measurements were performed at the Geochemistry and Isotope Geology Department, University of Göttingen, Germany.

Supplementary Data 2: Triple oxygen isotope compositions of magnetic and non-magnetic separates from magmatic rocks associated with the Kiruna IOA deposits

This table reports the $\delta^{17}O$, $\delta^{18}O$, and $\Delta'^{17}O$ values of mineral separates from porphyry rocks associated with the Kiruna IOA deposits, together with descriptions of rock type and sampling locations (SWEREF99 20 15). Samples were split into magnetic (magnetite-rich) and non-magnetic (silicate-rich) fractions using a hand magnet. The dataset also includes the calculated λ-values for each sample, representing the apparent slopes defined by the magnetic and non-magnetic fractions in linearised triple oxygen isotope space. All measurements were performed at the Geochemistry and Isotope Geology Department, University of Göttingen, Germany.

Supplementary Data 3: Electron microprobe analyses of apatite in magnetite-apatite rocks from Kiruna

This table provides electron-microprobe analyses (major and selected trace elements) of apatite from IOA rocks in the Kiruna district. Analyses were performed on a JEOL JXA-iHP200F at 15 kV and 20 nA using a focused beam at the University of Göttingen, Germany. Data were corrected using the XPP $\phi(\rho z)$ routine, and oxygen contents were calculated by stoichiometry.
