# Supplementary material for: Formation of iron oxide-apatite deposits triggered by magmatic assimilation of evaporitic sulfate
Source: Nat Commun. 2026 Jul 7;17:5930. doi: 10.1038/s41467-026-75189-0 (PMC13342607; doi:10.1038/s41467-026-75189-0)
Supplement: Supplementary file 5 — Transparent Peer Review file [file 41467_2026_75189_MOESM5_ESM.pdf]

Peer Review File

Formation of iron oxide-apatite deposits triggered by magmatic assimilation of evaporitic sulfate

Corresponding Author: Dr Stefan Peters

This file contains all reviewer reports in order by version, followed by all author rebuttals in order by version.

Version 0:

Reviewer comments:

Reviewer #1

(Remarks to the Author)
General comment:

The genesis of iron oxyde-apatite deposits is an intriguing petrological and gechemical subject of study, and the connection of these deposits to rare-earth elements occurence has provided additional interest on economic perspective. Different pieces of evidence support the involvement of sulphate in the evolution of magmas from which magnetite crystallized. However, large uncertainties exist about the mechanisms of formation, due to the lack of undigested residues in the magmas. Overall the process of sulphate assimilation into the magma is poorly understood; thus quantitative estimates of sulphate contribution to the formation of IOA-deposits is welcomed.

In their manuscript, the authors use high-precision triple oxygen geochemistry to trace the extent of sulphate assimilation by the magma associated to IOA-deposits, in order to understand if a genetic relationship can be established. The possibility to trace such an assimilation by triple oxygen is based on the distinct 17O-isotope anomaly in the sulphates, resulting from incorporation of atmospheric oxygen at the time of their formation. The O-isotope “connection” should be particularly relevant, provided the magnetite precipitation is expected upon melt oxidation after sulphate ingestion.

The manuscript is well written and the application of the triple oxygen systematics rigorous. The subject and the novel analytical application certainly fit with NC standards.
In addition, I found that the ultra-low D’17O values of magnetite and apatite provide a convincing evidence for melt assimilation of pre- and Cambrian sulphates, the latter characterized by having incorporated 17O of pre- and Cambrian atmosphere.
I wonder whether we could expect less negative D'17O-isotope values for younger IOA-deposits generated by magmas that assimilated younger (post 2.3 Ga, or even with lower atmospheric pCO2, post 34Ma) evaporites, or the D’17O values of sulphate can change only due to bacterial consuption.
Since apatite and magnetite are considered in isotopic equilibrium in the magma, it would be nice if the authors may further constrain their conclusions by showing if any covariation trend exist between apatite D’17O values and Cl/F/SO3 contents. The concentration of these elements should be enriched if magmatic assimilation of evaporitic material occurred. Any covariation trend diplayed by apatite chemical composition with O-isotope data would place a quite definitive constraint on IOA-deposit formation by evaporite assimilation in the magma.

In conclusion, this paper is a very nice gechemical study based on high-precison isotope investigation, and a nice example for future studies. Therefore I support the publication on NC after the minor integration suggested above.

Additional minor corrections shall be done at the lines:
-31: in the second term please report 18O instead of 17O;
-68: citation of Feng et al., 2021 is incomplete i the Refs;
-85: pls put the - range in parenthesis

I hope to see this manuscript published in Nature Communication soon.

Reviewer #2

(Remarks to the Author)
Summary

This manuscript presents new triple oxygen isotope ($\Delta^{17}O$) data from iron oxide-apatite (IOA) deposits mostly from the type-locality (Kiruna). The analytical methods appear to be robust, and the manuscript appropriately references previous works for methodological details. However, sample characterization (the spatial and petrographic descriptions) could be further developed to enhance clarity and robustness. The dataset provides valuable insights and builds on the authors' previous work (Peters et al., 2019), contributing to the ongoing discussion on IOA genesis. However, some interpretations may extend beyond the direct implications of the data.

Major Comments and Suggested Revisions

1. Context and Literature Review
The manuscript would benefit from a more comprehensive engagement with recent research on IOA deposits that supports a magmatic-hydrothermal origin. A significant body of literature over the past decade (e.g., Reich et al., 2022; Palma et al., 2021; Knipping et al., 2019; Simon et al., 2018; Ovalle et al., 2018; Knipping et al., 2015) provides important context that could strengthen the discussion. Incorporating these references would help to better situate the findings within the broader field.

2. Sample Classification and Geological Context
The relationship between the magnetite samples and the associated igneous units in the Kiruna district could be more clearly articulated. Providing additional spatial and petrographic context would improve clarity and strengthen the interpretations, particularly in relation to the multiple generations and types of magnetite present in IOA deposits.

3. Interpretation of Oxygen Isotope Data
The description of $\Delta^{17}O$ values could be refined for greater precision. The differentiation between “low” and “similar to crustal rocks” may be somewhat broad, as some non-Kiruna samples categorized as “low” appear to be more closely aligned with crustal values than with magnetite equilibrated with sulfate. A more detailed discussion of these isotope ratios would enhance the clarity and accuracy of the interpretations. Additionally, the potential role of magnetite equilibrating with fluids that exchanged oxygen with evaporites should be considered.

4. Genetic Implications and Interpretation Refinements
• The reported equilibrium temperatures of magnetite-apatite pairs align with values from IOAs in the Chilean belt (e.g., Rodriguez-Mustafa et al., 2020; Palma et al., 2020), which underscores the importance of integrating both igneous and magmatic-hydrothermal processes when discussing IOA formation.
• The higher $\delta^{18}O$ values from a couple of samples could be explained by other processes, thus the claim that the geological setting is “conceivable” for carbonate melt incorporation is not supported.
• The assumption that IOA-forming melts must be Fe-rich could be reconsidered in light of models (e.g., Knipping et al. 2015) that suggest hydrous-andesitic magmas can exsolve fluids with sufficient Fe to generate IOA deposits in the Chilean Belt. As this assumption is critical to the reaction the authors are proposing as the controlling factor in magnetite crystallization, the limitations of such assumptions should be discussed in the text.
• The interpretation of sulfate cycling at Grängesberg might need additional support, as an increase in $\delta^{18}O$ values would be expected as well if sulfate had undergone cycling.
• The interpretation that higher $\delta^{18}O$ values indicate carbonate assimilation may require additional discussion, as these values could also result from equilibrium with carbonate-equilibrated fluids.

Version 1:

Reviewer comments:

Reviewer #1

(Remarks to the Author)
I appreciate authors' responses. I agree with their interpretation of the data and I consider the manuscript ready to be published.

Open Access This Peer Review File is licensed under a Creative Commons Attribution 4.0 International License, which permits use, sharing, adaptation, distribution and reproduction in any medium or format, as long as you give appropriate credit to the original author(s) and the source, provide a link to the Creative Commons license, and indicate if changes were made.
In cases where reviewers are anonymous, credit should be given to 'Anonymous Referee' and the source.
The images or other third party material in this Peer Review File are included in the article's Creative Commons license, unless indicated otherwise in a credit line to the material. If material is not included in the article's Creative Commons license and your intended use is not permitted by statutory regulation or exceeds the permitted use, you will need to obtain permission directly from the copyright holder.
To view a copy of this license, visit https://creativecommons.org/licenses/by/4.0/

Response to Reviewer #1:

1.1 Since apatite and magnetite are considered in isotopic equilibrium in the magma, it would be nice if the authors may further constrain their conclusions by showing if any covariation trend exist between apatite D'17O values and Cl/F/SO3 contents. The concentration of these elements should be enriched if magmatic assimilation of evaporitic material occurred. Any covariation trend diplayed by apatite chemical composition with O-isotope data would place a quite definitive constraint on IOA-deposit formation by evaporite assimilation in the magma.

We appreciate the reviewer's suggestion and agree that a covariation between apatite $\Delta'^{17}O$ values and their Cl, F, and SO_3 concentrations would provide additional support for our hypothesis that apatite from Kiruna crystallised from silicate magmas that had assimilated evaporitic sulfate. Following this recommendation, we measured Cl, F, and SO_3 concentrations in apatite from Kiruna using EMPA. In these samples, we did not observe any covariation between $\Delta'^{17}O$ values and the measured Cl, F, or SO_3 concentrations. However, the absence of such a covariation does not, in our view, contradict our hypothesis. This is for three reasons:

1. Post-magmatic alteration of apatite compositions.
A detailed study of apatite from Kiruna has shown that Cl, F, and SO_3 concentrations were modified under greenschist-facies conditions after ore formation and therefore no longer reflect primary magmatic compositions[1].

2. Large natural heterogeneity in sulfate $\Delta'^{17}O$ values.
Evaporitic sulfate from a single outcrop can show $\Delta'^{17}O$ heterogeneity of several hundred ppm[2,3]. We therefore interpret the ~200 ppm range in magnetite $\Delta'^{17}O$ values in our Kiruna dataset as reflecting, at least in part, assimilation of sulfate with heterogeneous $\Delta'^{17}O$ values, rather than variable amounts of sulfate assimilation. Apatite $\Delta'^{17}O$ values likely record this heterogeneity as well.

3. Assimilation of sulfate does not necessarily affect Cl and F.
Magmatic assimilation of evaporitic sulfate alone (the carrier of low $\Delta'^{17}O$-oxygen) would not significantly change the magma's Cl or F concentrations; only assimilation of halite-bearing evaporites would affect the Cl concentration in the magma.

Additional scatter in a $\Delta'^{17}O$ versus Cl/F/SO_3 plot is also expected from other factors that influence Cl/F/SO_3 concentrations, such as source heterogeneity, variable degrees of fractional crystallisation, and magma degassing[4]. Even if apatite preserved pristine Cl, F, and SO_3 concentrations, a clear correlation with $\Delta'^{17}O$ values would therefore not necessarily be expected.

In the revised manuscript, the new EMPA data for apatite samples from Kiruna have now been included as a supplementary table S3.

In conclusion, this paper is a very nice gechemical study based on high-precison isotope investigation, and a nice example for future studies. Therefore I support the publication on NC after the minor integration suggested above.

1.2 Additional minor corrections shall be done at the lines:
-31: in the second term please report δ18O instead of δ17O;

We thank the reviewer for noticing this, and have now replaced $\delta^{17}O$ in the second term with $\delta^{18}O$

1.3 -68: citation of Feng et al., 2021 is incomplete i the Refs;

The citation has now been updated to:

Feng, D., Tütken, T., Löffler, N., Tröster, G., & Pack, A. Isotopically anomalous metabolic oxygen in marine vertebrates as physiology and atmospheric proxy. Geochimica et Cosmochimica Acta 328, 85-102 (2022).

1.4 -85: pls put the δ- range in parenthesis

Parentheses have now been added.

I hope to see this manuscript published in Nature Communication soon.

References

1. Harlov, D. E. *et al.* Apatite–monazite relations in the Kiirunavaara magnetite–apatite ore, northern Sweden. *Chem. Geol.* **191**, 47–72 (2002).
2. Crockford, P. W. *et al.* Triple oxygen isotope evidence for limited mid-Proterozoic primary productivity. *Nature* Preprint at https://doi.org/10.1038/s41586-018-0349-y (2018).
3. Crockford, P. W. *et al.* Claypool continued: Extending the isotopic record of sedimentary sulfate. *Chem. Geol.* **513**, 200–225 (2019).
4. Marks, M. A. W. *et al.* The volatile inventory (F, Cl, Br, S, C) of magmatic apatite: An integrated analytical approach. *Chem. Geol.* **291**, 241–255 (2012).

Response to Reviewer #2:

2.1 (…) The manuscript would benefit from a more comprehensive engagement with recent research on IOA deposits that supports a magmatic-hydrothermal origin. A significant body of literature over the past decade (e.g., Reich et al., 2022; Palma et al., 2021; Knipping et al., 2019; Simon et al., 2018; Ovalle et al., 2018; Knipping et al., 2015) provides important context that could strengthen the discussion. Incorporating these references would help to better situate the findings within the broader field.

We have now better incorporated a discussion of several of the suggested literature sources in the manuscript. We now refer to the review paper by Reich et al. (2022) when introducing the geological enigma around the formation of IOA deposits:

> *"Most proposed formation models involve either igneous or hydrothermal processes, or a combination of both[5]."*
> (Lines 19 – 20)

In the revised manuscript, we now also refer to the work by Simon et al. (2018) and Palma et al. (2021) when discussing the magnetite-apatite oxygen isotope equilibrium temperatures:

> *"Such temperatures are consistent with those of fluids that reacted with the magnetite-apatite assemblage of the Chilean Iron Belt (…)[6,7]".*
> (Lines 124 – 125)

The work by Ovalle et al. (2018) is now included in the discussion as well:

> *"The formation of SO_2 gas by massive assimilation of evaporitic sulfate would additionally have facilitated the explosive degassing of the silicate magmas, similar to what is seen for El Laco[8]"*
> (Lines 182-184)

We appreciate the reviewer's suggestion to also consider the genetic model proposed by Knipping et al. (2015, 2019) for the Chilean Iron Belt. However, the geological setting of Kiruna differs fundamentally from that of the Chilean Iron Belt. The Knipping et al. (2015, 2019) model relies on hydrous andesitic intrusions, high fluid/rock ratios, and focused Fe–Cl-rich brine flow, i.e, conditions that are not applicable to the Kiruna district. To maintain a clear focus on the processes relevant to Kiruna, we have therefore not expanded the discussion in this direction.

2.2 The relationship between the magnetite samples and the associated igneous units in the Kiruna district could be more clearly articulated. Providing additional spatial and petrographic context would improve clarity and strengthen the interpretations, particularly in relation to the multiple generations and types of magnetite present in IOA deposits.

For each sample that was studied, we have now added a petrographic description to Table S1. As many of the samples studied here for their triple oxygen isotope compositions were already described and studied by previous workers, we have also added references to these previous studies to the table, in order to enhance the compatibility of our new triple oxygen isotope data for these samples with previously published data and sample descriptions.

2.3 The description of $\Delta^{17}O$ values could be refined for greater precision. The differentiation between "low" and "similar to crustal rocks" may be somewhat broad, as some non-Kiruna samples categorized as "low" appear to be more closely aligned with crustal values than with magnetite

equilibrated with sulfate. A more detailed discussion of these isotope ratios would enhance the clarity and accuracy of the interpretations.

We thank the reviewer for this suggestion, and have now incorporated a section in the introduction that discussed the triple oxygen isotope compositions of crustal rocks for comparison. We now also quantified "low $\Delta'^{17}O$" as any value below -75 ppm (Lines 43 – 52):

> *"Much different than atmospheric O_2 and evaporitic sulfate, most crustal rocks, minerals, and fluids plot along an array in $\Delta'^{17}O$–$\delta^{18}O$ space (a terrestrial fractionation array or TFA)[9,10]. When interpreting triple oxygen isotope data of rock samples, this TFA provides a baseline for assessing whether rocks contain evaporite-derived oxygen or not. The $\delta^{18}O$ values of most magnetite samples from IOA deposits plot in an interval of the TFA with $\delta^{18}O$ values of ca. –1 to 5‰[11–16], for which the corresponding $\Delta'^{17}O$ range is poorly constrained. When comparing the triple oxygen isotope compositions of rock samples with the TFA, we therefore rely on the $\Delta'^{17}O$ values of rocks and minerals with slightly higher $\delta^{18}O$ values, i.e., between 5 and 10‰. These rocks typically have $\Delta'^{17}O$ values between –45 and –75 ppm[9,10,17]. Fluids with lower $\delta^{18}O$ values than the samples studied here, in contrast, have higher $\Delta'^{17}O$ values than –45.[9] Thus, the IOA rocks studied are considered anomalously low relative to the TFA if they have $\Delta'^{17}O$ values below –75 ppm."*

Additionally, the potential role of magnetite equilibrating with fluids that exchanged oxygen with evaporites should be considered.

We have now better evaluated the possibility that magnetite and apatite in our samples exchanged oxygen with low-$\Delta'^{17}O$ fluids in a new section of the discussion (Lines 123 – 142):

> *"Magnetite-apatite pairs in three samples from the Kiruna IOA assemblage record oxygen isotope equilibrium temperatures of 450 – 500 °C (Fig. 2). Such temperatures are consistent with those of fluids that reacted with the magnetite-apatite assemblage of the Chilean Iron Belt directly after ore formation had ceased[6,18]; however, for Kiruna, this temperature range is also similar to previously reported greenschist facies conditions (T = 300°C – 450°C) to which the IOA assemblage was exposed several millions of years after ore formation[1]. Moreover, several much younger episodes of hydrothermal overprints have been documented in the area[19,20]. The $\delta^{18}O$ values of magnetite and apatite in these particular samples were therefore possibly modified by isotopic exchange between magnetite and apatite during metamorphism or later hydrothermal events unrelated to the mineralisation process. Importantly, such exchange could have involved external fluids[1], raising the possibility that the anomalously low $\Delta'^{17}O$ values of our samples reflect equilibration with an evaporite-derived metamorphic fluid at high fluid/rock ratios, rather than showing a primary signature of mineralisation. We consider this scenario unlikely, because magnetite-apatite pairs from three different Kiruna mines yield indistinguishable equilibrium temperatures within < 50°C. Fluid-rich metamorphism, in contrast, would be expected to produce spatially heterogeneous temperatures reflecting variable fluid access and flow paths. If our magnetite-apatite pairs would indeed record equilibrium temperatures of metamorphic oxygen isotopic exchange,*

they would need to have either exchanged with fluids in a rock-buffered system or, alternatively, by solid-state diffusion only. Neither scenario would have significantly altered the Δ′^{17}O values of magnetite and apatite by more than ~20 ppm from their pristine compositions. We therefore conclude that the low Δ′^{17}O values in our samples must represent a primary feature of the Kiruna IOA mineralisation process, rather than a metamorphic or hydrothermal overprint. This interpretation is further supported by similarly low Δ′^{17}O values in IOA samples from other localities that will be discussed below."

2.4 The reported equilibrium temperatures of magnetite-apatite pairs align with values from IOAs in the Chilean belt (e.g., Rodriguez-Mustafa et al., 2020; Palma et al., 2020), which underscores the importance of integrating both igneous and magmatic-hydrothermal processes when discussing IOA formation.

We agree with the reviewer about the importance of discussing hydrothermal processes. In the revised manuscript, it is now pointed out that the equilibrium temperatures of magnetite-apatite pairs align with values from IOAs in the Chilean belt (Lines 124 – 125):

"Such temperatures are consistent with those of fluids that reacted with the magnetite-apatite assemblage of the Chilean Iron Belt directly after ore formation had ceased[6,18]."

And (Lines 201 – 202):

"These fluids then crystallised additional magnetite and apatite to form vein and disseminated ore types at lower temperatures as is possibly recorded by the δ^{18}O values of magnetite-apatite pairs in our samples."

The higher δ^{18}O values from a couple of samples could be explained by other processes, thus the claim that the geological setting is "conceivable" for carbonate melt incorporation is not supported.

We agree that our suggested geological setting does not support carbonate melt incorporation. Rather, we aim to carefully point out that our suggested geological setting would be consistent with the presence of carbonate melt inclusions in some IOA deposits[21]. We have not better clarified this issue by rewriting the sentence (Lines 193 – 195):

"Finally, the formation of anatectic sulfate-carbonate melts is consistent with our suggested geological formation setting as well, as well, and could plausibly have produced magnetite with the observed Δ′^{17}O range observed in the Kiruna samples (Fig. 4), aligning with evidence from fluid inclusions that such melts contributed to the formation of some IOA deposits[9–12]"

The assumption that IOA-forming melts must be Fe-rich could be reconsidered in light of models (e.g., Knipping et al. 2015) that suggest hydrous-andesitic magmas can exsolve fluids with sufficient Fe to generate IOA deposits in the Chilean Belt. As this assumption is critical to the reaction the authors are proposing as the controlling factor in magnetite crystallization, the limitations of such assumptions should be discussed in the text.

We appreciate the reviewer's comment and the suggestion to discuss the model proposed by Knipping et al. (2015) for the Chilean Iron Belt. However, the focus of our manuscript is the IOA

deposits of the Kiruna district. The Knipping et al. (2015) model relies on a set of geological conditions (hydrous andesitic intrusions, high fluid/rock ratios, and focused Fe–Cl-rich brine flow) that may be appropriate for the Chilean Iron Belt but are not applicable to the formation of IOA deposits in Kiruna. To clarify this distinction more explicitly, we have revised the manuscript to emphasise that our model refers specifically to the Kiruna-type IOA deposits when we discuss "iron-rich" silicate magmas (see, for example, Lines 195–196 in the revised version):

> *"**For Kiruna**, we propose that the assimilation of evaporitic sulfate by iron-rich silicate magmas formed the onset of high-temperature melt and fluid-driven mineralisation processes."*

The interpretation of sulfate cycling at Grängesberg might need additional support, as an increase in $\delta^{18}O$ values would be expected as well if sulfate had undergone cycling.

We agree with the reviewer that microbial sulfur cycling can raise sulfate $\delta^{18}O$ values, but this effect is not as efficient as the resetting of the $\Delta'^{17}O$ anomaly; moreover, processes other than microbial sulfate cycling can also influence sulfate $\delta^{18}O$. Therefore, microbial sulfur cycling does not generate a correlation between $\Delta'^{17}O$ and $\delta^{18}O$ values (also see the data compilation and discussion by Crockford et al. 2019)[3]. In fact, Proterozoic sulfate lacking the atmospheric $\Delta'^{17}O$ anomaly can still exhibit $\delta^{18}O$ values of ~10 ‰. In order to clarify this in the manuscripts, we have now added the following note (Line 210):

> *"(…) without a significant effect in sulfate $\delta^{18}O$ values"*

The interpretation that higher $\delta^{18}O$ values indicate carbonate assimilation may require additional discussion, as these values could also result from equilibrium with carbonate-equilibrated fluids.

Yes, we agree with the reviewer and have now added the sentence (Lines 231-232):

> *"Alternatively, the carbonate-derived oxygen component could have been derived from fluids that equilibrated with carbonate rocks."*

References

(numbering also applies to quotations from the main manuscript)

5. Reich, M. *et al.* Formation of iron oxide–apatite deposits. *Nature Reviews Earth & Environment 2022 3:11* **3**, 758–775 (2022).

6. Palma, G. *et al.* Thermal evolution of Andean iron oxide–apatite (IOA) deposits as revealed by magnetite thermometry. *Sci. Rep.* **11**, 1–9 (2021).

7. Ovalle, J. T. *et al.* Formation of massive iron deposits linked to explosive volcanic eruptions. *Sci. Rep.* **8**, 14855 (2018).

8. Sharp, Z. D., Wostbrock, J. A. G. & Pack, A. Mass-dependent triple oxygen isotope variations in terrestrial materials. *Geochem. Perspect. Lett.* **7**, 27–31 (2018).

9. Herwartz, D. Triple oxygen isotope variations in Earth's crust. *Rev. Mineral. Geochem.* **86**, 291–322 (2021).

10. Childress, T. *et al.* Triple oxygen (δ18O, Δ17O), hydrogen (δ2H), and iron (δ56Fe) stable isotope signatures indicate a silicate magma source and magmatic-hydrothermal genesis for magnetite orebodies at El Laco, Chile. *Economic Geology* **115**, (2020).

11. Troll, V. R. *et al.* Global Fe–O isotope correlation reveals magmatic origin of Kiruna-type apatite-iron-oxide ores. *Nat. Commun.* **10**, 1–12 (2019).

12. Peters, S. T. M. *et al.* Triple oxygen isotope variations in magnetite from iron-oxide deposits, central Iran, record magmatic fluid interaction with evaporite and carbonate host rocks. *Geology* **48**, 211–215 (2020).

13. Jonsson, E. *et al.* Magmatic origin of giant 'Kiruna-type' apatite-iron-oxide ores in Central Sweden. *Sci. Rep.* **3**, 1644 (2013).

14. Rhodes, a L. & Oreskes, N. Oxygen isotope composition of magnetite deposits at El Laco, Chile: Evidence of formation from isotopically heavy fluids. *Economic Geology Special Publication No. 7: Geology and Ore Deposits of the Central Andes* **7**, 333–351 (1999).

15. Nyström, J. O., Billström, K., Henríquez, F., Fallick, A. E. & Naslund, H. R. Oxygen isotope composition of magnetite in iron ores of the Kiruna type in Chile and Sweden. *GFF* **130**, 177–188 (2008).

16. Peters, S. T. M. *et al.* Tight bounds on missing late veneer in early Archean peridotite from triple oxygen isotopes. *Geochem. Perspect. Lett.* **18**, 27–31 (2021).

17. Tornos, F., Hanchar, J. M., Munizaga, R., Velasco, F. & Galindo, C. The role of the subducting slab and melt crystallization in the formation of magnetite-(apatite) systems, Coastal Cordillera of Chile. *Miner. Depos.* **56**, 253–278 (2021).

18. Billström, K., Bergman, S., Mark, C. & Barfod, D. N. New 40Ar/39Ar mineral ages of Archaean to early Proterozoic rocks in northern Sweden and implications for the 1.8–1.5 Ga tectonothermal history of northernmost Fennoscandia. *International Journal of Earth Sciences* **114**, 347–368 (2025).

19. Yan, S., Wan, B. & Andersson, U. B. Hydrothermal circulation at 1.8 Ga in the Kiruna area, northern Sweden, as revealed by apatite geochemical systematics. *Precambrian Res.* **395**, 107151 (2023).

20. Bain, W. M. *et al.* A fundamental role of carbonate–sulfate melts in the formation of iron oxide–apatite deposits. *Nat. Geosci.* **13**, 751–757 (2020).
